# Hydrogen Production from the LOHC Perhydro-Dibenzyl-Toluene and Purification Using a 5 µm PdAg-Membrane in a Coupled Microstructured System

**DOI:** 10.3390/ma13020277

**Published:** 2020-01-08

**Authors:** Alexander Wunsch, Tatjana Berg, Peter Pfeifer

**Affiliations:** Institute for Micro Process Engineering, Karlsruhe Institute for Technology, 76344 Eggenstein-Leopoldshafen, Germany; alexander.wunsch@kit.edu (A.W.); berg_tatjana@t-online.de (T.B.)

**Keywords:** LOHC, LOHC-Dehydrogenation, PdAg-membranes, micro reactor, hydrogen purification

## Abstract

Hydrogen bound in organic liquid hydrogen carriers (LOHC) such as dibenzyl-toluene enables simple and safe handling as well as long-term storage. This idea is particularly interesting in the context of the energy transition, where hydrogen is considered a key energy carrier. The LOHC technology serves as a storage between volatile energy and locally and timely independent consumption. Depending on the type of application, decisive specifications are placed on the hydrogen purity. In the product gas from dehydrogenation, however, concentrations of 100 to a few 1000 ppm can be found from low boiling substances, which partly originate from the production of the LOHC material, but also from the decomposition and evaporation of the LOHC molecules in the course of the enormous volume expansion due to hydrogen release. For the removal of undesired traces in the LOHC material, a pre-treatment and storage under protective gas is necessary. For purification, the use of Pd-based membranes might be useful, which makes these steps less important or even redundant. Heat supply and phase contacting of the liquid LOHC and catalyst is also crucial for the process. Within the contribution, the first results from a coupled microstructured system—consisting of a radial flow reactor unit and membrane separation unit—are shown. In a first step, the 5 µm thick PdAg-membrane was characterized and a high Sieverts exponent of 0.9 was determined, indicating adsorption/desorption driven permeation. It can be demonstrated that hydrogen is first released with high catalyst-related productivity in the reactor system and afterwards separated and purified. Within the framework of limited analytics, we found that by using a Pd-based membrane, a quality of 5.0 (99.999% purity) or higher can be achieved. Furthermore, it was found that after only 8 hours, the membrane can lose up to 30% of its performance when exposed to the slightly contaminated product gas from the dehydrogenation process. However, the separation efficiency can almost completely be restored by the treatment with pure hydrogen.

## 1. Introduction

The call for a change, caused by climate change, is gaining importance in our society. Mostly young people and scientists demonstrate for climate protection on a regular basis. Sustainable technologies and renewable energy sources are the key to a healthy ecosystem for future generations. Hydrogen plays an important role in this development, as the molecule can be produced and converted in a climate-friendly way—meaning without greenhouse gas emissions. With the use of renewable energy, hydrogen can be produced by the electrochemical splitting of water. Its potential as fuel is enormous; in the medium to long term, the mobility sector can be fed with hydrogen for applications such as passenger and freight transport by train or ship. However, hydrogen is usually transported and stored under high pressure or in liquid form. The volumetric energy density under atmospheric conditions is simply too low and therefore uneconomical. Hydrogen has a high-risk potential, since hydrogen can form flammable or even explosive mixtures with air over a wide concentration range [1].

**Figure 1:**
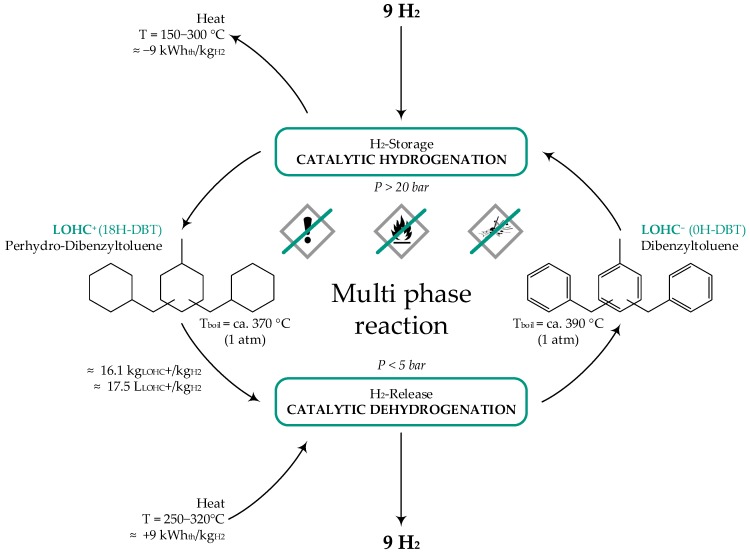
Scheme of the cycle of an organic liquid hydrogen carrier (LOHC) molecule, in this case dibenzyl-toluene, including its properties, adapted from [5].

The answer to this problem could be the so-called organic liquid hydrogen carriers (LOHC) technology [2]. With this approach, hydrogen is chemically bound in an organic, temperature-resistant liquid with a high density (up to a mass fraction of 6.2%). In this state, the loaded carrier can be stored or distributed without losses. If the hydrogen is needed, the carrier can be unloaded independently of time and location. Afterwards the LOHC material can be used for the next loading and unloading cycle (see Figure 1). The liquid used in this paper, namely dibenzyl-toluene, is well known as a heat-transfer oil, which is similar to conventional liquid fuel, with the benefit that it is flame-retardant. Furthermore the liquid has no hazard potential and is toxicologically harmless. All this advantages suggest that the technology will be widely accepted by the society [3,4]. 

**Figure 2:**
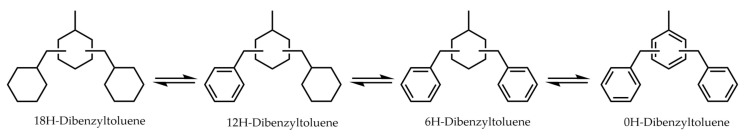
Schematic drawing of the LOHC system perhydro-dibenzyl-toluene/dibenzyl-toluene and its stable forms with different hydrogenation levels.

The fields of application for hydrogen are numerous—in addition to fuel cells, hydrogen is also used as a starting material in the chemical industry (e.g., in ammonia synthesis). Each application requires a defined hydrogen purity, which must be ensured at the end of the LOHC cycle. The release of hydrogen from the loaded LOHC (dibenzyl-toluene) takes place via a chemical reaction in the presence of a catalyst (usually Pt-based) at temperatures between 270–330 °C and low pressures. Two stable intermediates (12H-DBT, 6H-DBT) are formed on the reaction path to the completely unloaded form (0H-DBT). Those originate from the consecutive dehydrogenation of the C6 rings (see Figure 2). The LOHC is not an explicit compound, but a mixture of fully hydrogenated but also partially hydrogenated isomers as intermediates, and completely dehydrogenated C6 rings in one molecule. The general structures of the molecules do not change in the course of the reaction. [6,7]

During the reaction, the applied LOHC remains mostly liquid, so that a multiphase flow, consisting of the liquid LOHC and the gaseous hydrogen, leaves the reactor. Both phases are typically cooled down to near room temperature in the process and separated in a trap. So far, not much has been reported about the quality of the product gas. When comparing our data with the literature, it contains impurities from 100 to a few 1000 ppm, depending on the operating conditions [8]. The undesirable substances are CO, CO_2_, CH_4_ and higher, mostly cyclic hydrocarbons [9]. However, it has not yet been finally clarified how all these impurities are formed. Some byproducts are derived from the LOHC material production and/or are formed by the reaction. Oxygen-containing impurities are probably caused by atmospheric oxygen, the residual moisture of the LOHC material or the washing out of catalyst. Low boiling hydrocarbons may also be present in the starting material but can likely be formed by the decomposition of the LOHC molecules at elevated temperature. In order to ensure a high level of hydrogen purity, pre-treatment of the LOHC and post-treatment of the hydrogen are therefore necessary, as well as a low dehydrogenation temperature for avoiding further contaminants in the hydrogen and the LOHC. One advantageous possibility to avoid any contaminants in the hydrogen is the use of a Pd-based membrane. 

Despite the high costs for the material and the production of the membranes, palladium has unique and highly interesting properties in the field of hydrogen production and purification. Palladium has the highest solubility of hydrogen of all metals and allows the selective transport of hydrogen from a gas mixture through the material. The thinner the material, e.g., a membrane with a thickness of a few micrometers, the higher the flow rate. A defect-free membrane theoretically reaches infinite permselectivity. However, at temperatures below 293 °C, palladium and hydrogen form a mixed-phase region; hydrogen in the membrane lattice generates stress in the course of cooling down of the membrane, e.g. during shutdown of the separator, and promotes embrittlement of the membrane material. For this reason, pure Pd membranes can only be used at higher temperatures (>300 °C). Alloys can influence the properties of hydrogen separation and prevent the formation of different phases in the course of heating-up or cooling-down of the membrane under hydrogen atmosphere. The addition of silver in a certain ratio (23 mass%) can minimize the stress in the metal lattice, increase the robustness against mechanical and material influences and thus lower the operating temperature. [10,11,12,13,14,15,16,17] PdAg-membranes are therefore well suited for direct coupling with dehydrogenation reactors for the LOHC material system perhydro-dibenzyl-toluene/dibenzyl-toluene at the same temperature level. Depending on the desired catalyst mass-specific hydrogen release rate, 270 °C to 330 °C reaction temperature are favored for system operation. 

It has already been shown in previous studies that Pd-based membrane films integrated in microstructured apparatuses work more efficiently due to negligible concentration gradients, which typically occur in conventional reactor and thus limit the permeation flux. Microstructures also offer advantages for the reaction. The low heat transfer resistance ensures a sufficient heat supply of the strongly endothermic dehydrogenation reaction (71 kJ∙mol^−1^ per mole of H_2_) and good phase contacting of liquid and catalyst. In our previous work on this topic, we have already presented the multi-stage concept of reactor and membrane separator, which is also a topic of this work [18]. Within the present contribution, results from the real coupling of a radial flow microstructured reactor and membrane is investigated.

## 2. Materials and Methods 

### 2.1. Reactor

**Figure 3:**
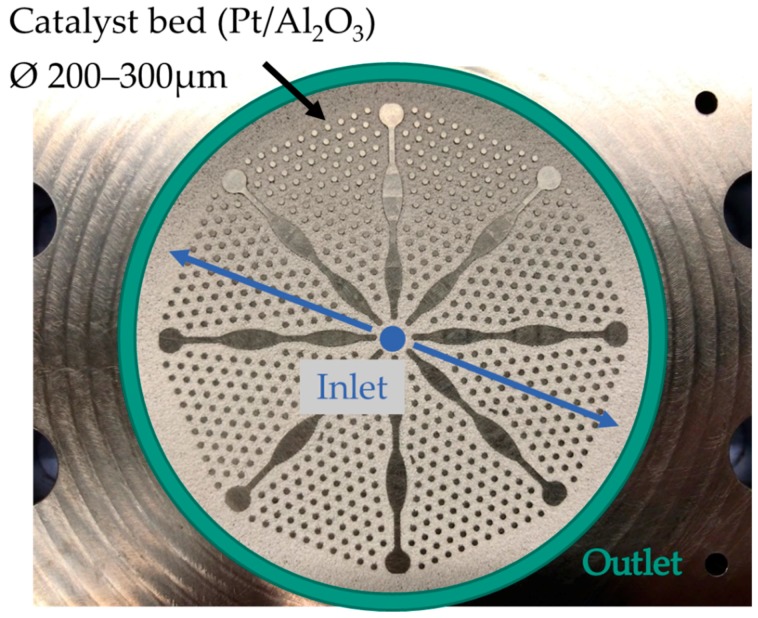
Picture of the microstructure filled with catalyst.

A microstructured radial flow reactor was used (see Figure 3). The microstructure consists of several circular sectors that are separated by fins and hexagonal distributed pins whose gaps are filled with catalyst. The LOHC enters in the middle and flows radially outwards, where the product mixture is collected in a circular ring channel below a circular metal filter, before leaving the reactor. A detailed description of the apparatus can be found in [18]. The catalyst used was provided by Clariant (Heufeld, Germany) and has a core-shell configuration. The carrier is porous Al_2_O_3_, while the shell consists of the active component platinum. The catalyst was tuned with sulfur due to improved activity and reduced side product formation [19]. For the experiments, the microstructure was filled with particles of the fraction of 200–300 µm without dilution; thereby a total catalyst mass of 1.51 g was integrated. The Pt loading is 0.3 mass%.

### 2.2. Membrane Separator

**Figure 4:**
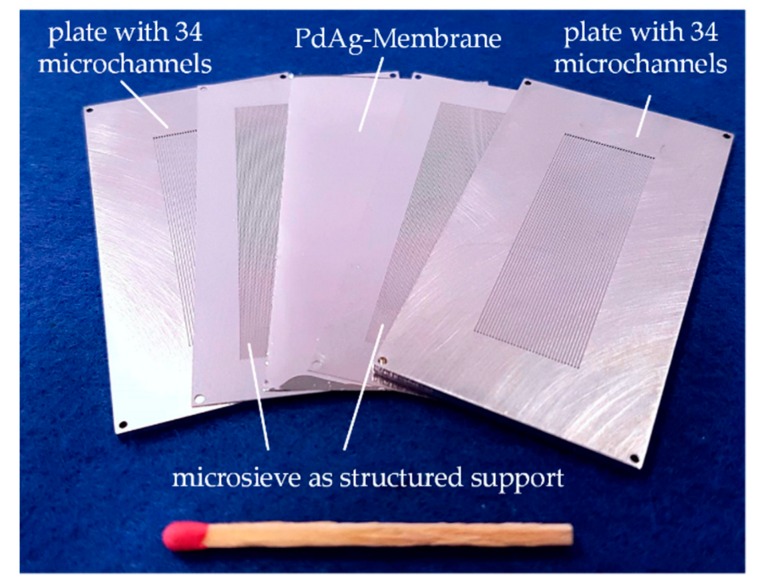
Structure of the microstructured membrane module. The used 5 µm PdAg membrane is mechanically stabilized by two micro sieves and integrated between two foils with micro channels. The stack is then bonded by laser welding [21].

The microstructured membrane separation module used (see Figure 4) has already been presented in previous work [18,20,21]. The module consists of a total of five elements—a 5 µm thick PdAg foil is mechanically stabilized with stainless steel micro sieves on each side, and placed between two foils with 17 micro channels (length × height × width: 4 cm × 300 µm × 500 µm) and welded together with laser technology. An accessible membrane area of 1.5 cm² was calculated while considering the area covered by the microsieve. The membrane was produced by magneton sputtering and provided by SINTEF (Oslo, Norway) [22].

### 2.3. Test Rig and Experimental Procedure

**Figure 5:**
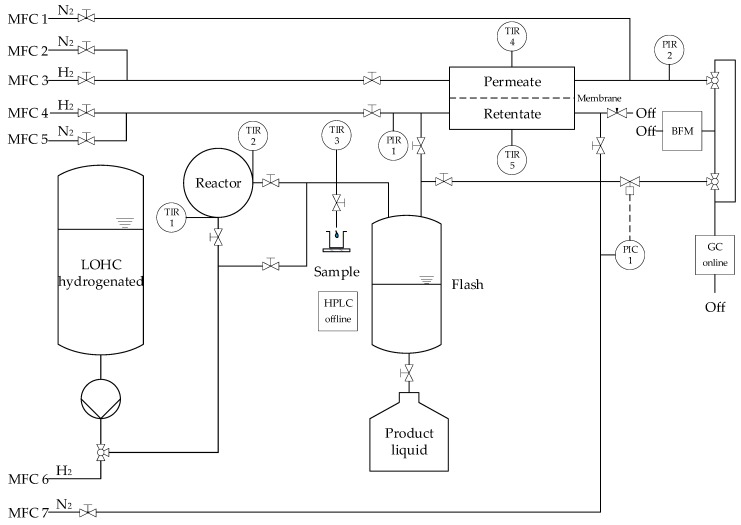
Flow diagram of the lab plant for the coupled release and purification of hydrogen.

Figure 5 shows a simplified process flow diagram of the test rig. The LOHC is pumped via a micro annular gear pump (MZR-2905, HNP Mikrosysteme GmbH, Schwerin, Germany) from the tank into the system. The liquid is heated and then fed into the reactor. Closely before and after the catalyst bed, the temperature is measured (TIR 1, TIR 2) and averaged for further calculations, although negligible temperature differences have been observed (less than 2 °C). The product flow is cooled down to ambient temperature (25 °C, TIR 3 is used for control) up to the sampling point, where liquid is collected and analyzed via high performance liquid chromatography (HPLC) with a refractometer as detector. The gas and liquid phases are separated from each other in a tank as phase separator. The product gas can either be directed to the membrane or directly to the gas chromatograph (GC) or bubble meter for analysis. The membrane separates the product into retentate and permeate, which can be measured separately or in mixed state in the GC or bubble column. The bubble meter serves as a control of the calculated fluxes in the GC. The reactor and membrane separator are electrically heated separately via heating cartridges. The temperature of the membrane was measured outside, directly at the membrane module, both on the retentate and permeate side (TIR 4, TIR 5), and averaged (less than 1 °C difference). The gas supply during start-up and shut-down phase—as well as for the reduction of the catalyst or pure gas experiments with the membrane—is provided by mass flow controllers (MFC 2-6). MFC 1 and MFC 7 were used to add nitrogen as internal standard (ISTD) for GC analysis. N_2_ from MFC 7 was used to provide stable pressure by a pressure retention valve (Flowserve GmbH, Essen, Germany), because the membrane area could be so large that most hydrogen will be permeated, depending on the operating conditions. N_2_ by MFC 7 was also required for initial pressure build-up.

#### 2.3.1. Membrane Characterization

At the beginning, the membrane was heated to a temperature of 325 °C in a nitrogen atmosphere. A leak test was carried out at 4 bar pressure difference; no leakage through the membrane was found. Afterwards, the membrane was exposed to hydrogen at ambient pressure. After 24 h, the pressure on the retentate side was increased to 4 bar and held for 48 h. After that, permeation experiments were carried out under a constant hydrogen flow of 400 mL∙min^−1^ using a bubble meter to measure retentate and permeate flux. The temperature was varied between 300–340 °C and the pressure between 1 and 4 bar respectively. The flux corresponds to the product of permeance П and pressure difference between retentate and permeate side (see Equation (1)). The Sieverts exponent n was determined from these experiments.

(1) $${\dot{F}}_{H_{2}}\left[\frac{mol}{m^{2}\times s}\right]=\Pi \times \left(p_{H_{2},Ret}^{n}-p_{H_{2},Perm}^{n}\right)$$

The permeance П results from the ratio of the material-related permeability Q and the membrane thickness s. The relationship between permeability and temperature can be described using the Arrhenius approach (see Equation (2)) [10,23].

(2) $$\Pi \left[\frac{mol}{m^{2}\times s\times Pa^{n}}\right]=\frac{Q}{s}=\frac{Q_{0}\times \exp\left(-\frac{E_{A,M}}{RT}\right)}{s}$$

#### 2.3.2. Coupling of Reactor and Membrane Separator

After reduction of the catalyst at 330 °C for 24 h, the pressure was increased, the LOHC supply was started (23 g∙h^−1^) and product liquid was periodically collected and analyzed until the catalyst reached a steady state. The LOHC mass flow corresponds to a modified residence time of approx. 40 kg_Cat_∙m^−3^∙h. To calculate the modified residence time, the applied catalyst mass is divided by the volume flow of the liquid at reaction temperature (see Equation (3)). The starting material (Hydrogenious Technologies) had a degree of dehydrogenation (DoDH) of 3.82% and has not been pretreated with regard to impurities. Based on the initial composition, the mixture density is determined as a function of the reaction temperature. The density correlations can be taken from [24].

(3) $$\tau_{mod}\left[\frac{kg_{Cat}}{m^{3}}h\right]=\frac{M_{cat}}{{\dot{V}}_{LOHC}\left(T_{R}\right)}$$

Before coupling, the membrane was operated at a constant temperature of 300 °C and supplied with hydrogen from gas bottles in identical amount of the measured product gas flow at reaction pressure. This avoided a pressure pulse on the membrane during the upcoming coupling step. The product gas flow was then directed to the membrane and the hydrogen supply was stopped at the same time. In the coupled state, permeate and retentate flows were analyzed alternatingly every 30 min over a period of almost 8 h. Overnight, reactor and membrane were decoupled and the reaction pressure was changed; the membrane itself was again fed with hydrogen from bottles. The next day, the procedure was repeated. In total, experiments at pressures of 2, 3 and 4 bar were applied. Before and after each coupling experiment, a reference point for hydrogen permeation was set to see a possible loss of membrane separation performance.

### 2.4. Analysis of Product Liquid

The product liquid was analyzed offline by HPLC-RI combination (Series 200, PerkinElmer Rodgau, Germany). The methodology was taken from [25]. A hexyl-phenyl column (Phenomenex Luna 5 µm phenyl hexyl 100 Å, 250 × 4.6 mm) with acetone/water 96/4 vol% as solvent was used. Peaks of the fully loaded form (18H-DBT) and the fully unloaded form (0H-DBT) were calibrated with a standard of the pure components and the peaks of the intermediates (6H-DBT, 12H-DBT) were linearly interpolated between 18H-DBT and 0H-DBT, as they are not available in pure form. The interpolation is well justified because the refractive index changes proportionally to the degree of hydrogenation (DoH) [24]. The DoH describes the actual amount of bound hydrogen atoms in relation to the maximum possible quantity of hydrogen atoms that can be bound (see Equation (4)). For the calculation of the DoH, the molar fractions of the differently saturated LOHC compounds are multiplied with the bound hydrogen atoms and summarized. The degree of dehydrogenation (DoDH) corresponds directly to the DoH, as it can be seen in Equation (5).

(4) $$DoH\;\left[-\right]=\frac{\sum {\tilde{x}}_{i}\times n_{H,i}}{n_{H,total}}=\frac{{\tilde{x}}_{0H-DBT}\times 0+{\tilde{x}}_{6H-DBT}\times 6+{\tilde{x}}_{12H-DBT}\times 12+{\tilde{x}}_{18H-DBT}\times 18}{18}$$

(5) DoDH [−]=1−DoH

A decisive value for the process is the productivity of the total amount of Pt in the catalyst (see Equation (6)), which describes the ratio between the mass flow of hydrogen released and the mass of the catalytically active component (mass of catalyst multiplied with the platinum mass fraction).

(6) $$P\left[\frac{g_{H_{2}}}{g_{Pt}\times min}\right]=\frac{{\dot{M}}_{H_{2}}}{M_{Cat}\times x_{Pt}}$$

### 2.5. Analysis of Product Gas

The product gas was analyzed in a gas chromatograph (6890A, Agilent Technologies Deutschland GmbH, Germany) with FID and TCD. A non-polar column (HP-1, ID 320 µm, 30 m) was used to separate organic compounds, while a molecular sieve (HP-Plot, 5 Å, ID 320 µm, 15 m) was used for the permanent gases. Argon 5.0 was used as carrier gas. N_2_, H_2_, O_2_, and CO were calibrated on the TCD and CH_4_ on the FID. The organic by-products from the reaction, other than methane, were treated as a group and related to a standard of methyl-cyclohexane. This is justified, because mainly C_7+_ derivatives are formed from the decomposition of the LOHC molecules. Due to the increasing intensity of the FID signal with the number of C atoms, the analysis represents the worst case as it overestimates the real impurities as soon as larger molecules than C_7_ are present. For the calculation of absolute volume flows, 20 mL∙min^−1^ N_2_ as Internal Standard (ISTD) were added via MFC 7 for the retentate and product gas and MFC 1 for the permeate.

## 3. Results and Discussion

### 3.1. Membrane Characterization

**Figure 6:**
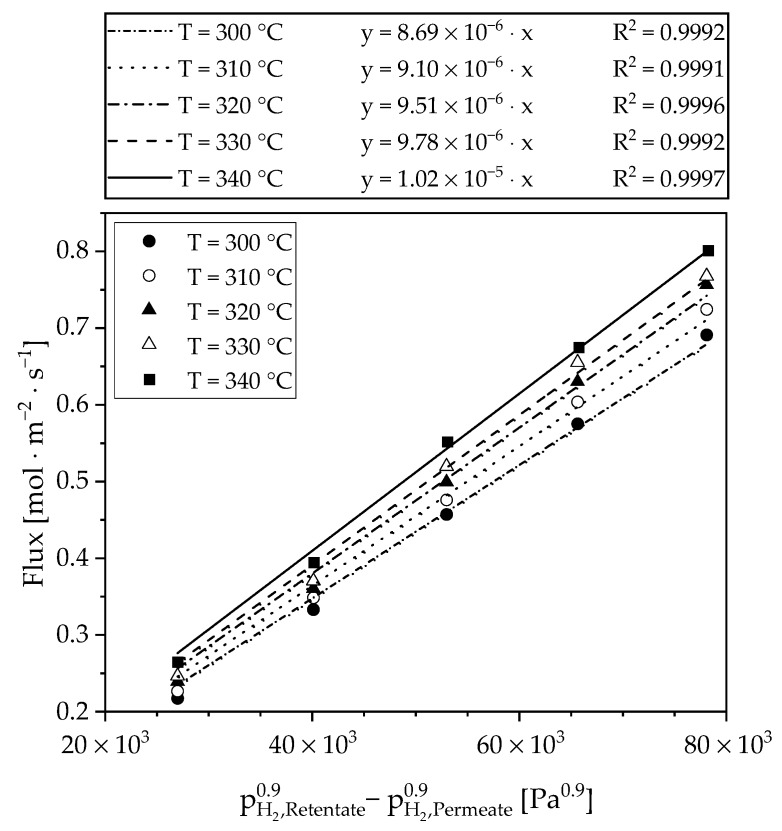
Sieverts plot of the investigated 5 µm thick PdAg membrane. The fitting of the measured data resulted in a Sieverts exponent of n = 0.9.

**Figure 7:**
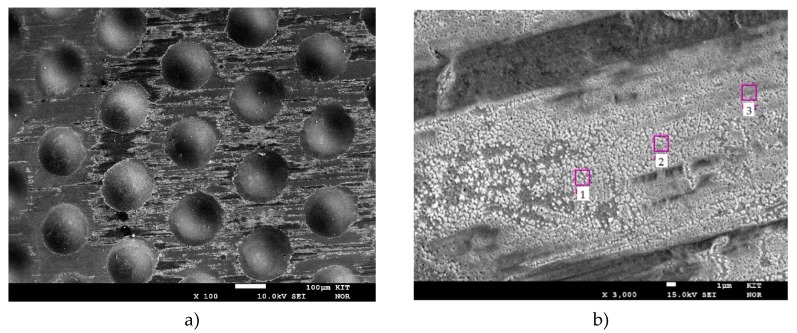
Microprobe images of the membrane surface after permeation tests: (**a**) 100 times enlarged; (**b**) 3000 times enlarged with marked analysis points, where iron is found.

The results of the pressure and temperature variation are shown in Figure 6—the flux is plotted as a function of the driving force. The permeance according to Equation (1) can be taken from the slope of the linear fit at the corresponding temperature. The classical square root law of Sieverts describes solid diffusion as a limiting step in the solution diffusion mechanism. However, with this assumption (n = 0.5) it was not possible to sufficiently describe the measured data (R^2^ ≈ 0.954). The Sieverts exponent was then fitted to a value of n = 0.9 using the least squares method. This high value proves that another step determines separation process. Since desorption limitations play practically no role at these temperatures, the separation is limited by the adsorption of hydrogen at the membrane surface. One explanation for this is the low membrane thickness of 5 µm, which reduces the importance of the diffusion path and increases the influence of sorption and surface reaction steps. However, the value is very high compared to previous work with the same type of membrane yielding n = 0.7 [20]. After the experiment, the surface was examined with a microprobe. The images can be seen in the Figure 7 and indicate a deposit partially covering the membrane surface. An elemental analysis (see points in Figure 7b) has shown that this deposit is iron based. This could originate from the microsieve contact. Adding the micro sieve without a diffusion barrier layer (DBL) seems to lead to intermetallic diffusion despite low temperatures. This finding strengthens the assumption of the limitation by surface phenomena. The activity of the surface palladium for dissociative hydrogen adsorption is lowered in presence of other materials like iron. This could explain the factor of 0.9.

**Figure 8:**
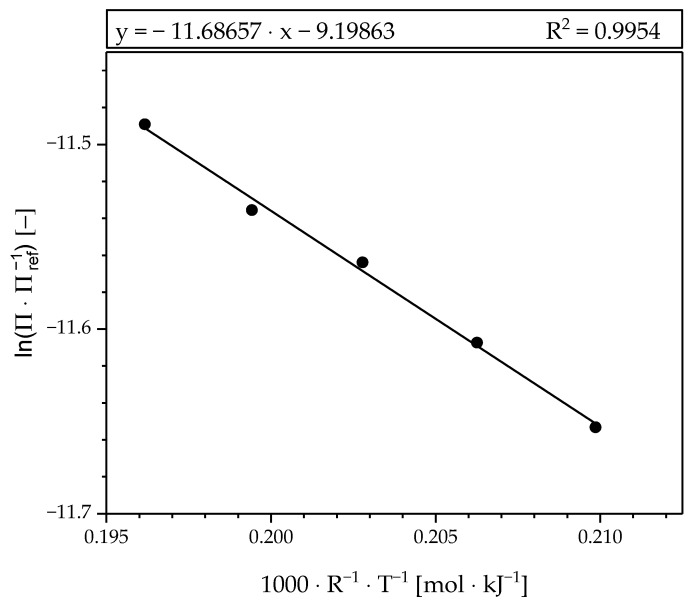
Arrhenius plot of the fitted permeances from the Sieverts plot for n = 0.9.

The Arrhenius plot for estimating the temperature dependence of the permeance (Equation (2)) is shown in Figure 8. The permeances obtained are plotted in logarithmic form over the temperature. The slope of the fitted line equals the activation energy (E_A,M_), while the pre-exponential factor can be calculated from the intercept. The curve represents the measured values with high quality. Table 1 shows the fitted values at n = 0.9 and, for comparison to literature, at n = 1

The fit of the permeance based on n = 1 is provided, since it is more realistic than applying n = 0.5. Chen et. al. [26] have applied palladium-based composite membranes, whose hydrogen permeation is clearly controlled by surface phenomena and can be described with n = 1. Their membranes are characterized by a particularly high permeability, which was confirmed by a literature comparison [26]. 

We compared the permeances obtained by the fit at n = 1 with the data provided by Chen et al. [26] in Table 2 at a temperature of 350 °C. It can be seen that our determined permeance values correspond very well to their given values. This good agreement may confirm the assumption that surface phenomena limit the transport through the membrane.

### 3.2. Coupling of Reactor and Membrane Separator

**Figure 9:**
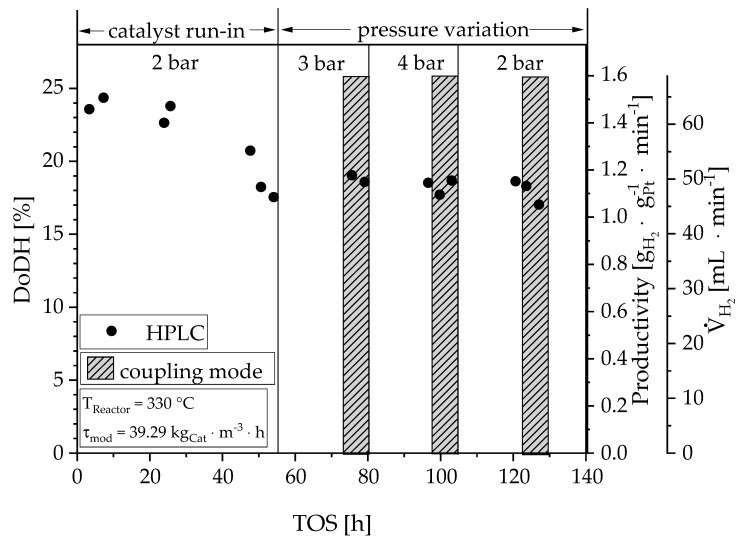
Degree of Dehydrogenation, productivity and produced hydrogen flow, determined by HPLC, during the entire test duration of 140 hours. The shaded areas represent the periods in the coupled mode.

For the coupling of the membrane with the reactor, the membrane characterized in the previous section was replaced by a fresh one from the same batch. Figure 9 shows the degree of dehydrogenation (DoDH), as well as the calculated parameters: catalyst-related productivity and standard volume flow as a function of the time on stream. The vertical lines mark the pressure in the corresponding time period. The shaded areas indicate the time intervals in which the product gas was fed to the membrane separation system (coupled state). After an initial two days, a stationary state was reached—during this time the degree of dehydration dropped from 24% to 18 %. In the course of the following pressure variation, the degree of dehydrogenation remained constant. A high productivity of 1.15 g_H_2__∙g_Pt_^−1^∙min^−1^ was achieved. Out of 23 g∙h^−1^ LOHC approx. 3 NL∙h^−1^ were released. Both the HPLC and GC measurements (for the sake of clarity not mentioned further) confirm these findings. Regardless of the fact that the hydrogen partial pressure has a positive influence on the stability of the catalyst, it was expected that the degree of dehydrogenation decreases with increasing pressure due to thermodynamics (confirmed in other experiments [27,28]). Thus, this observation might be explained by limitation, by reaction kinetics, or by a pressure-dependent residence time change in the reactor. The hydrogen bubbles from the reaction change their volume depending on the pressure. The larger the bubbles (the lower the pressure), the more the liquid is pushed out of the small reaction chamber. As a result, the actual residence time levels out a higher intrinsic reaction rate at lower pressure.

#### 3.2.1. Membrane Degradation and Regeneration Behavior

**Figure 10:**
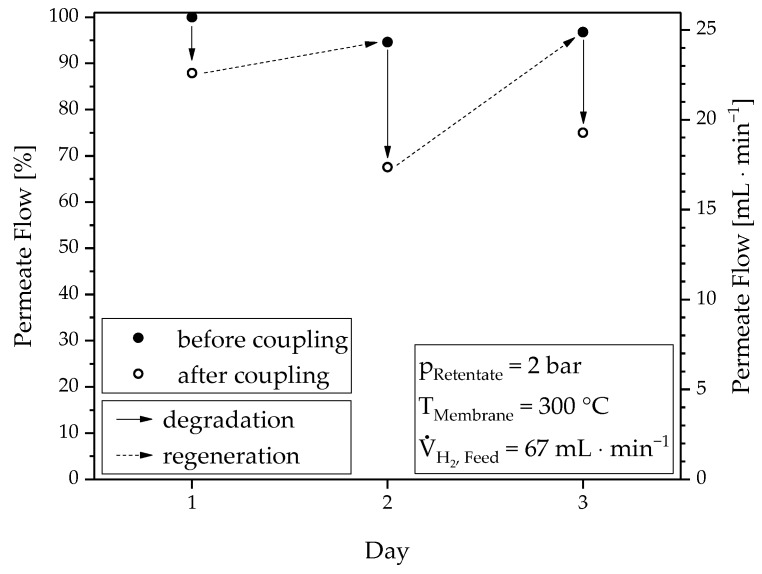
Degradation and regeneration behavior of the membrane: Hydrogen flow before and after each coupling experiment at constant conditions (2 bar, 300 °C and 67 NmL∙min^−1^).

The influence of the contaminated product gas on the separation performance by the permeation reference point before and after the coupling experiment is obvious from the determined H_2_ flow rates in Figure 10. The normalized value of 100% at day 1 indicates the flow at the time when the membrane had not yet had contact with the product gas. Starting from this point, the separation efficiency decreased in the course of each day, by a maximum of approx. 30% (see day 2). The performance loss on day 3 was less than on day 2, although the operating time was longer. This behavior was probably caused by the higher operating pressure, as more impurities adsorb on the membrane surface. However, the original separation efficiency was almost completely restored when flushing the membrane overnight with 450 mL∙min^−1^ bottle gas. Neither the time nor the volume flow have been varied so far. Thus, the required regeneration time and flushing gas amount needs further study. Nevertheless, it is remarkable that the low impurities in the product gas caused a relatively strong reduction in hydrogen flow. However, since this process is reversible, this phenomenon supports the observation that the permeation process is influenced by surface phenomena (n = 0.9) in the given membrane arrangement. The organic by-products, mainly aromatic compounds, compete with hydrogen for the adsorption spots on the membrane surface; also liquid formation could occur on the surface at high separation efficiency. The reduction in the effective membrane area for hydrogen separation results in a decreasing flux. During the regeneration process, the impurities are removed from the surface area.

#### 3.2.2. Impurity Measurements

**Figure 11:**
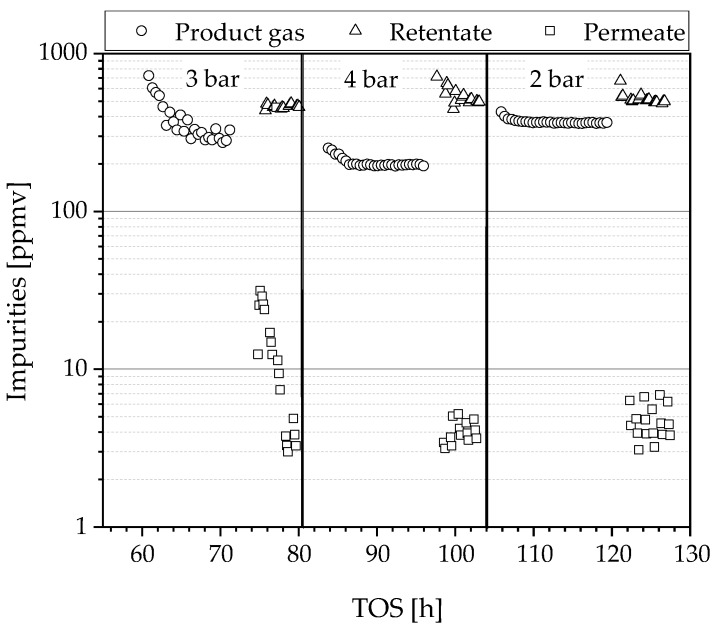
Impurities of the product gas, retentate and permeate measured by GC in ppmv and logarithmic form over the time on stream.

The impurities in the product gas, retentate and permeate were determined by gas chromatography, as described in Chapter 2.5, and plotted over the time on stream in Figure 11. In the product gas, impurities vary between 200–400 ppmv and decrease with higher reaction pressure. An explanation is that these impurities are condensed in higher percentages as the total pressure was increased. Another possible explanation from a reaction point of view is the higher hydrogen partial pressure, which causes more hydrogen to adsorb on the catalyst causing inhibition of by-product formation. Contrary to the literature, no CO could be detected [8,9]. Possible explanations are the increasing methanation activity (formation of methane via hydrogenation of CO) with increasing pressure and/or the poor detection limit of the GC for CO. Independent of the pressure applied, the impurities in the hydrogen could be reduced to 3–7 ppmv, which corresponds to a quality of 5.0 (>99.999%). Between 74–78 h a concentration higher than 10 ppmv was measured in the permeate at 3 bar, which can be explained by residual contamination of the GC sampling loop. The same GC was used for the analysis of both streams, permeate and retentate; an indication is the ubiquitous trend to lower values over time on stream. To minimize this problem, the flushing time between the measurements was increased between the measurements of 3 bar and 4 bar. However, it could not be guaranteed that all residues were removed. Since only aromatic compounds were detected in the permeate, it can be assumed that these are mainly contaminants of the sample loop and that the purity is actually higher than measured.

## 4. Conclusions

Overall, the results suggest that LOHC technology, microstructure devices and Pd-based membranes are an efficient combination for the on-site supply of pure hydrogen. 

First, the highly stable catalyst-related hydrogen productivity of 1.15 g_H2_∙g_Pt_^−1^ min^−1^ at 330 °C and 4 bar(a) from the non-pretreated LOHC material should be underlined. Due to the high ratio of surface area to volume, the microstructure ensures a sufficiently high heat input to the strongly endothermic reaction. Practically no temperature gradient was measured along the reactor length. 

Second, it has been proven that operation at a moderate pressure of 4 bar is efficient, since the degree of dehydrogenation in the applied operation conditions has not been reduced by the pressure increase as expected from thermodynamics. Moreover, the impurities in the product gas can be reduced to approx. 200 ppmv by higher pressure in order to ensure better recyclability of the LOHC.

Third, the high flowrates of hydrogen permeate obtained from the PdAg membrane fit quite well to the dehydrogenation conditions. Nevertheless, due to the small membrane thickness of 5 µm, the adsorption process has a high impact. Even small amounts of contaminants on the membrane surface affect the hydrogen flux. This process of surface blocking is reversible and may be easily circumvented by back-flushing of the membrane unit.

The purified gas contains impurities in the range of 3–7 ppmv, equal to hydrogen 5.0 according to the limited gas analytics available. To be sure, the analysis methodology must be considered critically. The detected compounds indicate the presence of contamination in the GC periphery and sample loop. Under this assumption, the real concentrations would be overestimated. For further efforts an improvement of the analytics is planned. Specific regulations on hydrogen 5.0 or fuel cell quality exist which include also further parameters such as inert gases and other contaminants which could not be investigated yet.

In the future, experiments consisting of a longer time on stream are planned, to provide information about the deactivation behavior of the catalyst and the longevity of the membrane. Furthermore, experiments on LOHC dehydrogenation and purification will be carried out in a combined membrane micro reactor.

## Figures and Tables

**Table 1:** Measured activation energy and pre-exponential factor from membrane characterization experiments.

	n=0.9 [−]	n=1 [−]
$E_{A,M}\;\left[kJ\cdot mol^{-1}\right]$	11.69±0.46	11.67±0.46
$Q_{0}\;\left[mol\cdot m^{-1}\cdot Pa^{-n}\cdot s^{-1}\right]$	$5.05\times 10^{-10}\pm 0.49\times 10^{-10}$	$1.33\times 10^{-10}\pm 0.13\times 10^{-10}$

**Table 2:** Comparison of the determined permeances with a fit at n = 1 with the data provided in the work of Chen et al. [26]. Only membranes with high permeance are considered.

Membrane	Thickness [μm]	$Permeance\;\left[mol\cdot m^{-2}\cdot Pa^{-1}\cdot s^{-1}\right]$
Pd/PSS [26]	6.93	$3.93\times 10^{-6}$
Pd/Cu/PSS [26]	7.03	$2.78\times 10^{-6}$
Pd/PSS [26]	6.63	$2.40\times 10^{-6}$
$Pd_{77}Ag_{23}\;\left[This\;work\right]$	5	$2.83\times 10^{-6}$
